# Roles of microglial mitophagy in neurological disorders

**DOI:** 10.3389/fnagi.2022.979869

**Published:** 2022-08-10

**Authors:** Yang Liu, Miao Wang, Xiao-Ou Hou, Li-Fang Hu

**Affiliations:** ^1^Department of Neurology and Clinical Research Center of Neurological Disease, The Second Affiliated Hospital of Soochow University, Suzhou, China; ^2^Jiangsu Key Laboratory of Neuropsychiatric Diseases and Institute of Neuroscience, Soochow University, Suzhou, China

**Keywords:** microglia, mitochondria, mitophagy, neurological disorders, neuroinflammation

## Abstract

Microglia are the resident innate immune cells in the central nervous system (CNS) that serve as the first line innate immunity in response to pathogen invasion, ischemia and other pathological stimuli. Once activated, they rapidly release a variety of inflammatory cytokines and phagocytose pathogens or cell debris (termed neuroinflammation), which is beneficial for maintaining brain homeostasis if appropriately activated. However, excessive or uncontrolled neuroinflammation may damage neurons and exacerbate the pathologies in neurological disorders. Microglia are highly dynamic cells, dependent on energy supply from mitochondria. Moreover, dysfunctional mitochondria can serve as a signaling platform to facilitate innate immune responses in microglia. Mitophagy is a means of clearing damaged or redundant mitochondria, playing a critical role in the quality control of mitochondrial homeostasis and turnover. Mounting evidence has shown that mitophagy not only limits the inflammatory response in microglia but also affects their phagocytosis, whereas mitochondria dysfunction and mitophagy defects are associated with aging and neurological disorders. Therefore, targeting microglial mitophagy is a promising therapeutic strategy for neurological disorders. This article reviews and highlights the role and regulation of mitophagy in microglia in neurological conditions, and the research progress in manipulating microglial mitophagy and future directions in this field are also discussed.

## Introduction

Mitochondria are the hubs of nutrient metabolism and the powerhouse of the cell that produces a large amount of adenosine triphosphate (ATP) through oxidative phosphorylation. Intriguingly, mitochondria also serve as a source of reactive oxygen species (ROS) due to the reaction between oxygen and leaked electrons from the mitochondrial electron transport chain. ROS act as signaling molecules in various physiological processes or cause severe damage to cells when overwhelmingly accumulated. ROS directly impair mitochondrial proteins, lipids and DNA (mtDNA), resulting in mitochondrial dysfunction, which in turn produces more ROS and forms a vicious cycle. The accumulation of dysfunctional mitochondria is associated with cancer, aging and neurodegeneration (Sun et al., 2016; Srivastava, 2017). In addition, some developmental processes, such as erythrocyte differentiation and maturation, rely on the elimination of mitochondria (Mortensen et al., 2010). Therefore, the removal of damaged or redundant mitochondria is critical for the development, normal function and survival of cells.

Mitophagy refers to an intracellular process that selectively eliminates redundant or damaged mitochondria by autophagosome sequestration and delivers the cargo to lysosomes for degradation. This term was first described by Scott and Klionsky (1998) and formally proposed by Lemasters (2005), highlighting the selective feature of this process. In the past two decades, an increasing number of studies have demonstrated the roles of mitophagy in the quality control and biogenesis of mitochondria and revealed the contribution of mitophagy defects to aging, cancer and a variety of diseases.

## Regulation mechanism of mitophagy

Mitophagy is an evolutionarily conserved process. Recent studies have revealed an intricate crosstalk between signaling and mitophagy initiation pathways and identified the well-conserved machinery of mitophagy from yeast to mammals. In 2009, two independent groups led by Yoshinori Ohsumi and Daniel J Klionsky simultaneously and consistently reported an indispensable role of Atg32, an outer mitochondrial membrane (OMM) protein, as the receptor of mitophagy in yeast through the interaction with Atg8 and Atg11 (Kanki et al., 2009; Okamoto et al., 2009). Subsequent studies also found a cooperative regulation of Atg32 expression and phosphorylation for mitophagy in yeast (Montenarh, 2010; Aoki et al., 2011; Kondo-Okamoto et al., 2012). The initial studies in yeast laid the groundwork for our understanding of mammalian mitophagy.

**FIGURE 1:**
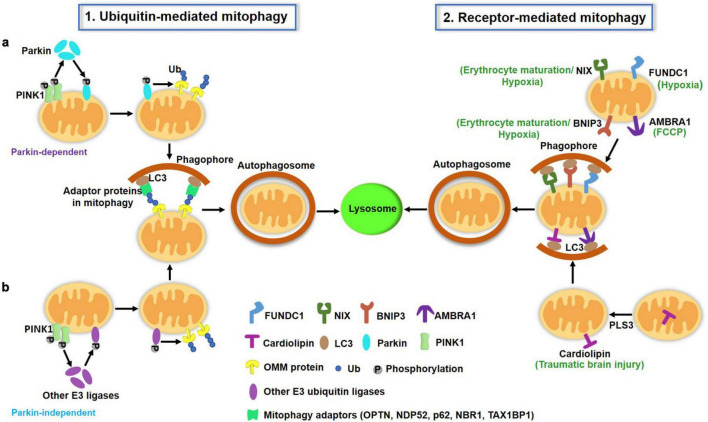
A schematic illustration of two major pathways of mitophagy in mammalian cells. (1) Ubiquitin (Ub)-mediated mitophagy pathways. PINK1 accumulates on the OMM of depolarized mitochondria and undergoes auto-phosphorylation. **(a)** PINK1 then recruits cytosolic Parkin translocation to the OMM and activates this E3 Ub ligase by phosphorylation, which ubiquitinates several OMM proteins such as mitofusin, VDAC and Miro. PINK1 also directly phosphorylates the preexisting Ub on the OMM. Ub is also able to recruit and activate Parkin. The actions of PINK1 and Parkin cooperatively lead to the polyUb process of damaged mitochondria, which can be recognized by mitophagy adaptors (p62, OPTN, NDP52, NBR1, TAX1BP1) to mediate mitochondria sequestrated by autophagosomes. **(b)** Apart from Parkin, other E3 ubiquitin ligases [ARIH1, seven *in absentia* homolog (SIAH)-1 and RNF34, etc.] have been reported to mediate mitophagy in a PINK1-dependent manner. (2) Receptor-mediated mitophagy. OMM proteins including FUNDC1, AMBRA1, BNIP3L/Nix, BNIP3 serve as mitophagy receptors under specific conditions (hypoxia, erythrocyte maturation, and toxin exposure). These receptors directly bind to LC3 *via* the LIR motif. Additionally, a few IMM proteins such as cardiolipin and prohibitin 2 can translocate to the OMM under certain conditions, where they also serve as mitophagy receptors and promote mitophagy through their LIR to interact with LC3 on the autophagosomes. OMM, outer mitochondrial membrane; IMM, inter mitochondrial membrane; LIR, LC3-interacting region.

In mammals, there are two major pathways of mitophagy: the ubiquitin (Ub) Pink/Parkin pathway and the receptor-dependent pathway, as illustrated in Figure 1.

### Ubiquitin-mediated mitophagy pathway

Ub-mediated mitophagy, mainly triggered by PTEN-induced putative kinase protein 1 (PINK1) and the E3 Ub ligase Parkin, has been intensively and extensively studied *in vitro*, and is well reviewed elsewhere (Eiyama and Okamoto, 2015). Under normal conditions, PINK1 is translocated to the inner mitochondrial membrane (IMM), where it is cleaved by presenilin-associated rhomboid-like (PARL) protein (Meissner et al., 2015), thereby maintaining a low basal level on healthy, polarized mitochondria. Once mitochondria are depolarized, PINK1 cannot be translocated into the IMM and thus accumulates on the OMM, where it forms homodimers and undergoes autophosphorylation. PINK1 then recruits Parkin from the cytosol to the OMM surface and activates this E3 Ub ligase by phosphorylation. Activated Parkin then ubiquitinates its substrates and leads to the formation of Ub chains (Sha et al., 2010). PINK1 also phosphorylates preexisting Ub molecules on the mitochondrial surface (Ordureau et al., 2014). The actions of PINK1 and Parkin both contribute to the polyUb process of damaged mitochondria in a positive feedback fashion.

Ubiquitinated mitochondria can be recognized by adaptor proteins, including p62/SQSTM1, OPTN, NDP52, NBR1, and TAX1BP1 (Geisler et al., 2010; Heo et al., 2015), which can bind to ubiquitinated cargoes through the Ub-associated (UBA) domain and LC3-tagged autophagosomes *via* the LC3-interacting region (LIR). Thus, adaptor proteins are essential for the process of Ub-mediated mitophagy through assisting in mitochondrial sequestration and engulfment by autophagosomes. Whether these adaptor proteins have unique or redundant effects on mitophagy under specific conditions remains unclear. An elegant study from Richard J Youle’s lab revealed the central yet redundant roles of NDP52 and OPTN in PINK1- and Parkin-mediated mitophagy in HeLa cells (Lazarou et al., 2015). Researchers have also identified an important role of TANK binding kinase 1 (TBK1) in the regulation of these adaptor proteins and the mitophagy process (Heo et al., 2015; Richter et al., 2016).

PINK1/Parkin-mediated mitophagy is typically studied *in vitro* and represents a major pathway in defense against mitotoxicity. Its role *in vivo* remains to be clarified. A recent study provided the first *in vivo* evidence that loss of *Pink1* did not alter basal mitophagy in tissues or cells of high metabolic demand including microglia, using the mito-QC reported mouse model (McWilliams et al., 2018). *Pink1* KO mice exhibited indistinguishable basal mitophagy in metabolically active tissues compared with *WT* mice. The findings highlight the possibility that other unidentified pathways may participate in the basal mitophagy process in these tissues. In support of this, neither *PINK1 or Parkin KO* mice recapitulated overt neurodegenerative phenotypes under normal conditions (Perez and Palmiter, 2005; Kitada et al., 2007).

The formation of a polyUb chain on the OMM is essential for PINK1/Parkin-mediated mitophagy. Overexpression of the deubiquitinating enzymes (DUBs) USP30 and USP35 inhibited Parkin-mediated mitophagy. In contrast, interfering with these two DUBs promoted Parkin-mediated mitophagy (Wang et al., 2015). Different DUBs may regulate mitophagy in different manners. USP15 antagonizes Parkin-mediated mitochondrial ubiquitination and thus mitophagy (Cornelissen et al., 2014). Alternatively, USP8 preferentially removed non-canonical K6-linked Ub chains from Parkin and thereby blocked its recruitment to depolarized mitochondria and mitophagy activity (Durcan et al., 2014). It is noteworthy that Parkin-independent mitophagy may also exist (Villa et al., 2017; Oshima et al., 2021). Other E3 Ub ligases, such as ARIH1, seven *in absentia* homolog (SIAH)-1, and RNF34, have been reported to mediate mitophagy in a PINK1-dependent manner (Szargel et al., 2016; Villa et al., 2017; He et al., 2019). Therefore, future work is expected to fully understand the roles of E3 ligases or DUBs in Ub-mediated mitophagy in mammals.

### Receptor-mediated mitophagy pathway

Receptor-mediated mitophagy is another important mechanism for the clearance of damaged or redundant mitochondria. Several receptors, including FUN14 domain-containing 1 (FUNDC1), Autophagy and Beclin 1 Regulator 1 (AMBRA1), BCL2/adenovirus E1B 19-kDa-interacting protein 3-like (BNIP3L/NIX) and BCL2/adenovirus E1B 19-kDa-interacting protein 3 (BNIP3), and Prohibitin 2 (PHB2), have been identified for this process. The receptor-mediated mitophagy pathway does not require Ub signaling. Rather, the receptors directly bind autophagy-related proteins. These mitophagy receptors share a common feature that they contain a conserved LIR domain, which allows them to interact with LC3. This interaction is modulated by the phosphorylation and protein abundance of receptor molecules.

Most of these mitophagy receptors are OMM proteins. Intriguingly, the mitophagy receptor often functions uniquely in specific conditions. For example, FUNDC1 acts as a receptor in hypoxia-triggered mitophagy (Liu et al., 2012). Under normal conditions, FUNDC1 is phosphorylated by Src, which weakens its interaction with LC3. Upon hypoxia, the phosphatase PGAM5 dephosphorylates FUNDC1 at Ser13 and enhances its interaction with LC3 and thereby mitophagy (Chen et al., 2014). The structural basis for the phosphorylation regulation of FUNDC1 LIR as a molecular switch for mitophagy has been identified (Kuang et al., 2016). The E3 ligase membrane associated RING-CH5 (MARCH5) also regulates FUNDC1 and plays a fine-tuning role in hypoxia-induced mitophagy (Chen et al., 2017). Nix (also called Bnip3L) functions as a selective mitophagy receptor during erythrocyte maturation (Sandoval et al., 2008; Novak et al., 2010). *Nix-deficient* mice developed anemia and decreased erythrocyte maturation with mitochondrial retention. A homologous protein of Nix, BNIP3, also serves as a receptor in hypoxia-induced mitophagy. The phosphorylation of BNIP3 at the Ser17 and Ser24 sites enhanced its LIR interaction with LC3 and mitophagy (Zhu et al., 2013). The kinase(s) responsible for BNIP3 phosphorylation remain unclear. Additionally, researchers identified AMBRA1 as a powerful receptor that is crucial for both Parkin-dependent and -independent mitophagy in response to trifluoromethoxy carbonylcyanide phenylhydrazone (FCCP)-induced mitochondrial uncoupling (Strappazzon et al., 2015).

In addition, two IMM proteins, cardiolipin and prohibitin 2 (PHB2), were reported to mediate the turnover of unwanted or damaged mitochondria *via* mitophagy (Chu et al., 2013; Wei et al., 2017). Chu et al. reported that rotenone, 6-hydroxydopamine and other pro-mitophagic stimuli caused cardiolipin translocation from the IMM to the OMM, where it served as an “eat-me” signal for the recognition and engulfment of damaged mitochondria by autophagosomes. Phospholipid scramblase-3 (PLS3) is responsible for cardiolipin externalization during this process. Another study reported that the hexameric intermembrane space protein nucleoside diphosphate kinase-D (NDPK-D) can bind cardiolipin and facilitate its redistribution to the OMM and thus mitophagy initiation (Kagan et al., 2016). Cardiolipin-directed mitophagy acted as an endogenous neuroprotective process against traumatic brain injury (TBI) and behavioral deficits in rats. Knockdown of *cardiolipin synthase or PLS3* markedly reduced TBI-induced mitophagy (Chao et al., 2019). Similarly, a recent study identified another IMM protein, PHB2, as a mitophagy receptor that can bind to LC3 upon mitochondrial depolarization and protease-dependent OMM rupture (Wei et al., 2017). This process is required for Parkin-induced mitophagy in mammalian cells and for paternal mitochondria clearance after embryonic fertilization in *C. elegans*.

### Other mitophagy pathways

Other mitophagy pathways have been reported but remain less well understood. For example, Sentelle et al. reported a new receptor function of ceramide in mitophagy. Ceramide stress triggered lethal mitophagy by anchoring LC3B-II autophagosomes to mitochondrial membranes upon Drp1-mediated mitochondrial fission (Sentelle et al., 2012).

Taken together, distinct signaling pathways have been revealed to mediate or regulate the mitophagy processes in mammals. Although the mitophagy pathways have been intensively studied *in vitro* for the clearance of depolarized mitochondria, further studies are still needed to validate the *in vivo* roles of these mitophagy-relevant pathways using recently established mitophagy reporter mouse models (Sun et al., 2015; Williams et al., 2017). Further, little is known about the machinery for basal mitophagy, especially *in vivo*. This also needs to be investigated.

## Neuronal mitophagy in the brain

The brain is the organ with the highest energy demand in the body and accounts for 20% of the total oxygen consumption to supply ATP for intensive neuronal activities. As postmitotic cells, neurons are highly dependent on mitochondrial quality control and network homeostasis. Maintaining a pool of healthy mitochondria through mitophagy-mediated removal of damaged mitochondria is particularly important for neuronal function and survival. Using the *in vivo* mitophagy reporter *mt-Keima* transgenic mice, researchers found significant variations in basal mitophagy activity in neurons of different brain regions. The dentate gyrus, lateral ventricle and Purkinje fiber cells showed a relatively higher level of neuronal basal mitophagy, whereas a lower level was reported in the striatum, cortex and substantia nigra (Sun et al., 2015). Mitophagy defects in neurons are associated with aging and neurodegeneration, which has been extensively studied and reviewed elsewhere (Martinez-Vicente, 2017; Doxaki and Palikaras, 2020). However, the roles and regulation of mitophagy in glial cells, such as microglia in particular, are less studied but are increasingly gaining attention. In this article, we will focus on the role of microglial mitophagy in the brain during health and diseases, and the potential strategies for microglial mitophagy modulation will also be summarized and discussed.

## Mitophagy and innate immunity

**FIGURE 2:**
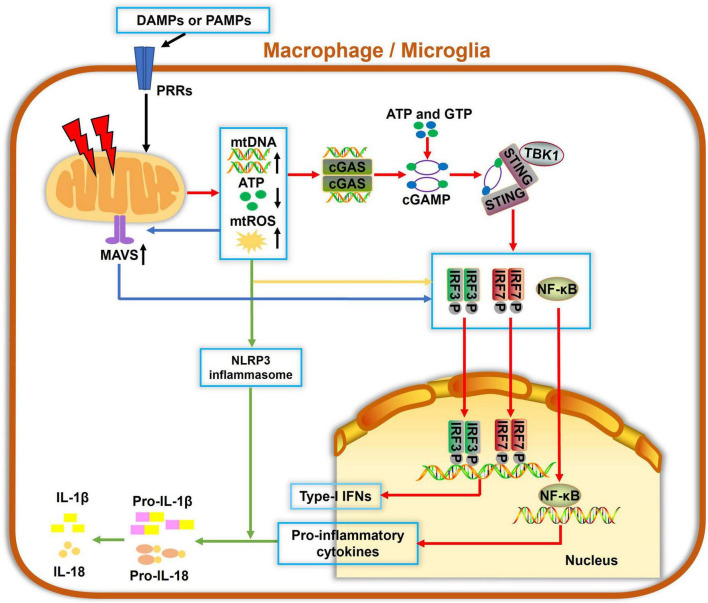
Mitochondria involvement in innate immune responses in macrophage/microglia. Damage-associated molecular patterns (DAMPs) and pathogen-associated molecular patterns (PAMPs) can be recognized by pattern recognition receptors (PRRs) to cause mitochondrial damage, which leads to increased mitochondrial reactive oxygen species (mtROS) formation, mitochondrial DNA (mtDNA) release, and decreased ATP generation. Cyclic guanosine monophosphate-adenylate synthetase (cGAS) is a cellular DNA sensor that primarily recognizes double-stranded DNA, i.e., mtDNA, using ATP and GTP as substrates to produce the second messenger cGAMP, which then binds to and trigger stimulator of interferon genes (STING) oligomerization. Activated STING recruits TBK1 and activate downstream IRF3, IRF7, or nuclear factor-κB (NF-κB), ultimately inducing the expressions of type-I interferons (IFNs) and proinflammatory cytokines. Also, mtDNA and mtROS are canonical activators of the NLRP3 inflammasome and IL-1β secretion. Additionally, mitochondrial antiviral signaling (MAVS) protein is a RIG-like receptor (RLR) localized on the OMM. mtROS accumulation potentiates the MAVS downstream cascade, which also contributes to activations of NF-κB, IRF3, and IRF7, leading to a pronounced generation of cytokines and IFNs. Collectively, damaged mitochondria serve as hubs of multiple signaling cascades in facilitating innate immune responses in macrophages and microglia.

Mitochondria are not only essential for energy supply in cells but also regulate the innate immune response to infectious and sterile insults. The initiation of innate immunity may be mitochondria-independent; however, mitochondria can serve as a signaling platform to facilitate immune responses, as summarized in Figure 2. Several molecules, such as ATP, N-formyl peptides or mitochondrial DNA (mtDNA), mitochondrial ROS (mtROS) and cardiolipin, can be released from compromised mitochondria under stressful conditions. They act as damage-associated molecular patterns (DAMPs) and can be sensed by pattern recognition receptors (PRRs), which are highly expressed by innate immune cells. These events often activate immune cells and result in the release of cytokines or chemokines, yielding inflammatory responses. For example, mtROS and cardiolipin were reported to activate the NLRP3 inflammasome and IL-1β secretion (Tschopp and Schroder, 2010; Shimada et al., 2012). mtDNA and mtROS also trigger mitochondrial antiviral signaling (MAVS) and the cGAS-STING pathway, which drives the production of type I interferons and other cytokines, resulting in a strong inflammation (Seth et al., 2005; Liu et al., 2015).

Mitophagy enhancement *via* genetic approaches or small molecules was shown to relieve mtROS accumulation and mtDNA release and, more importantly, inhibit the secretion of inflammatory factors *in vitro* and *in vivo* (Suen et al., 2010; Sliter et al., 2018; Fang et al., 2019b; Qiu et al., 2022). Conversely, autophagy-deficient macrophages showed an accumulation of defective mitochondria, accompanied by NLRP3 activation and IL-1β secretion (Saitoh et al., 2008; Nakahira et al., 2011). Remarkably, mitophagy is also modulated by inflammation. NLRP3 and AIM2 inflammasome activation was reported to cause caspase-1-dependent mitochondrial impairment and mitophagy blockade in bone marrow-derived macrophages (BMDMs) (Yu et al., 2014). Interestingly, activation of the classic proinflammatory transcription factor NF-κB resulted in increased expressions of p62/SQSTM1, which is recruited to damaged mitochondria and responsible for the mitophagy process. This NF-κB-p62-mitophagy pathway prevented the accumulation of damaged mitochondria and excessive IL-1β-mediated inflammation in LPS-stimulated macrophages, orchestrating a self-limiting response and favoring tissue repair (Zhong et al., 2016). Mitophagy also affects the phagocytosis of macrophages. These findings strongly suggest a complicated interaction between mitochondria and innate immunity in macrophages. Damaged mitochondria may function as signaling platforms in initiating innate immune responses. Conversely, inflammation has a differential effect on mitophagy-triggered clearance of dysfunctional mitochondria, which may be context dependent.

## Mitochondrial homeostasis and microglia

Microglia are tissue-resident macrophages constituting 5–10% of cells in the central nervous system (CNS). They serve as the first line of immune defense. Microglia originate from early erythromyeloid precursor cells in the yolk sac and migrate into the brain at embryonic day 9.5. However, they exhibit unique features that are distinct from BMDMs. Microglia have a longer half-life that reaches approximately 15 months in mice (Fuger et al., 2017). They persist and self-renew in the CNS throughout the life. Recent studies have revealed a robust capacity for microglial repopulation in microglia-depleted brain regions (Huang et al., 2018). Hence, microglia share the same origin as peripheral macrophages but have unique features different from their sibling cells.

As resident innate immune cells, microglia survey and maintain the homeostasis of the CNS with rapid process extension and retraction. They act as sensors and rapidly respond to various pathological stimuli. Once activated, ramified microglia transform into amoeboid morphology with enlarged soma, shorter and less branched processes, and also release a large number of cytokines/chemokines (Ghosh et al., 2018). The highly dynamic nature of microglia determines this population of cells in high energy demand in both physiological and pathological situations. Mitochondria are energy sources and metabolic centers in cells. Homeostasis of the mitochondrial network structure, which is determined by a balance of fission and fusion, greatly affects cellular function and survival. Metabolic reprogramming and regulation of gene expression by histone lactylation, a posttranslational modification by the metabolic byproduct lactate, are associated with microglia/macrophage polarization and intimately linked to several inflammation-related diseases (Baik et al., 2019; Zhang et al., 2019). Excessive mtROS generation was found in hyperactivated primary microglia and BV-2 cells (Ye et al., 2017) and was demonstrated to induce microglial polarization toward the M1 proinflammatory status (Zhou et al., 2018). Dysregulated mitochondrial fission was observed in inflamed microglia (Park et al., 2016; Katoh et al., 2017), whereas mitochondrial division inhibitor 1 (mdivi-1) and *dynamin-related protein 1 (Drp1)* knockdown inhibited mitochondrial fission, mtROS generation and proinflammatory mediators in LPS-stimulated microglia (Park et al., 2013). The natural compound atractylenolide III was reported to suppress neuroinflammation and protect against brain ischemia by inhibiting JAK2/STAT3-dependent mitochondrial fission in microglia (Zhou et al., 2019). In summary, disruption of mitochondrial homeostasis contributes to microglia-mediated inflammation and *vice versa*.

## Microglial mitophagy and neurological disorders

Microglia-mediated inflammation is one of the pathogenic factors in neurodegeneration and other neurological disorders. Environmental and genetic factor-associated damage or homeostatic disruption of mitochondria is also linked to these diseases. As an effective avenue to remove damaged mitochondria, mitophagy plays a critical role in regulating inflammation, phagocytosis and other processes in microglia. However, the role of microglial mitophagy in health and disease is less reviewed.

### Alzheimer’s disease

AD is the most common neurodegenerative disease and is characterized by progressive cognitive impairment and unique pathological changes, including extracellular amyloid β (Aβ) plaques and neurofibrillary tangles of hyperphosphorylated tau protein. To date, the pathogenesis of AD remains elusive.

The accumulation of impaired mitochondria is a hallmark of AD brains in patients and mouse models (Kerr et al., 2017). There are sophisticated connections between mitochondrial impairment and typical AD pathological changes. The expression of several mitochondrial dynamics-related proteins, including Drp1, Fis1, Mfn1, Mfn2, and OPA1, was abnormally changed in the brains of AD patients (Manczak et al., 2011). Aβ can disrupt the homeostasis of the mitochondrial structure and network, resulting in abnormal mitochondrial fission and damage (Wang et al., 2009). Tau hyperphosphorylation and tangle formation impairs the axonal transport of mitochondria and lead to axonal degeneration (Shahpasand et al., 2012). Furthermore, mitochondrial dysfunction contributes to AD pathology, which is well summarized and reviewed (Chakravorty et al., 2019). Therefore, it is proposed that promoting the clearance of defective mitochondria may become a potential approach for AD therapy. This hypothesis is supported by Khandelwal et al. (2011) study reporting that lentivirus-mediated *Parkin* overexpression enhanced mitophagy and reduced Aβ in 3XTg-AD mice. Consistently, mitophagy deficits were observed in AD-induced pluripotent stem cell (iPSC)-derived neurons, several transgenic animal models and patient brains of AD (Fang et al., 2019b). The researchers found a 60% decrease in mitophagy, coinciding with more damaged mitochondria, in microglia from the hippocampus of AD mice compared to WT mice. These changes were alleviated by the treatments with two screened mitophagy inducers, urolithin A (UA) and actinonin (AC). Increased microglial engulfment of Aβ plaques was observed following AC and UA treatment, indicating that mitophagy induction enhanced the efficiency of Aβ plaque phagocytosis by microglia, although the mechanisms remain unclear. Moreover, UA and AC consistently reduced the proinflammatory cytokines IL-6 and TNF-α, as well as NLRP3 inflammasome activation, and UA produced an additional increase in the anti-inflammatory cytokine IL-10 level in the hippocampal tissues of transgenic APP/PS1 mice. These effects were verified in isolated microglia, confirming that UA-modulated inflammatory response was mitophagy-dependent because *Pink1* depletion eliminated the UA-induced inhibition of TNF-α. In the study, Fang et al. demonstrated that restoration of mitophagy by UA, AC, or NAD^+^ supplementation mitigated Aβ and tau pathologies and reserved memory impairment in both transgenic tau nematodes and AD mouse model.

Recently, an unbiased proteomic study reported that orally administered melatonin for 1 month obviously affected the protein expression patterns in 5 × FAD mice, with less effect in WT mice. Bioinformatic and biochemical studies revealed that melatonin treatment ameliorated mitophagy deficits, microglial activation and other AD pathological and cognitive changes in 5 × FAD mice, all of which were reversed by chloroquine cotreatment. The findings implicate that mitophagy enhancement, at least in part, contributes to the neuroprotection of melatonin treatment in these AD mice (Chen et al., 2021). Nevertheless, the cellular roles of melatonin’s protective functions, either neuron- or glia-derived or even both, warrant further study, because melatonin receptors are also enriched in microglia and responsible for its anti-inflammatory properties (Gu et al., 2021).

In summary, microglial mitophagy defects are implicated in AD pathogenesis, in addition to neuronal mitophagy impairment. Enhancement of microglial mitophagy not only helps to resolve neuroinflammation but also improves phagocytosis and clearance of Aβ plaques. Further studies are required to identify the pathogenic factors responsible for the mitophagy deficits in microglia and clarify whether it is merely a consequence of other pathologic events in AD.

### Parkinson’s disease

Parkinson’s disease (PD) is the most common neurodegenerative movement disorder, pathologically characterized by dopaminergic neuron (DA) losses in the midbrain and α-synuclein-enriched Lewy body formation. Its etiology is still unknown and is assumed to be associated with genetic and environmental factors, as well as aging (Schapira et al., 2017). Mitochondrial dysfunction is implicated as a pivotal pathogenic factor in both familial and sporadic PD (Kalia and Lang, 2015).

Early studies found a dramatic decrease in complex I activity in the substantia nigra homogenate of PD patients compared with healthy controls (Schapira et al., 1990). MPTP, a byproduct of synthetic heroin that elicits PD-like symptoms in heroin abusers and a commonly used neurotoxin in remodeling PD, also inhibits the activity of complex I in the mitochondrial respiratory chain (Desai et al., 1996). Several changes related to mitochondrial dysfunction, such as decreased ATP levels, abnormal mtDNA release, mtROS accumulation, as well as altered mitochondrial structure and dynamics, have been reported in PD animal and cellular models (Chu et al., 2013; Rani and Mondal, 2020). More importantly, several PD-related proteins, such as Parkin, SNCA, DJ-1, LRRK2, UCHL-1, PINK1, vacuolar protein sorting 35 (VPS35), and HtrA2, participate in the regulation of mitochondrial homeostasis, and their mutants lead to mitochondrial impairment. As mentioned above, PINK1 and parkin function within the same mitophagy pathway, and play critical roles in the removal of damaged mitochondria. Strikingly, *PINK1-* and *parkin-*knockout mice did not show robust signs of PD (Perez and Palmiter, 2005; Kitada et al., 2007). Using mito-QC as an *in vivo* mitophagy reporter, a recent study demonstrated that basal mitophagy in mouse tissues with high metabolic demand such as microglia and PD-relevant mesencephalic DA neurons, occurred independently of PINK1, as loss of *Pink1* did not have any obvious effect on basal mitophagy in these cells/tissues (McWilliams et al., 2018). This indicates that other yet-to-be identified pathways may orchestrate mammalian mitochondrial integrity in a context-dependent fashion. Consistently, an article published in Nature 2018 reported a strong inflammatory phenotype in both *PINK1- and parkin*-deficient mice following exhaustive exercise and in Parkin^–/–^; mutator mice with accumulated mutations in mtDNA (Sliter et al., 2018). This finding supports a role for PINK1/Parkin-mediated mitophagy in restraining innate immunity, especially when challenged.

Several natural compounds were reported to enhance microglial mitophagy and suppress PD-related neuroinflammation. For instance, andrographolide inhibited NLRP3 inflammasome activation through triggering parkin-mediated mitophagy in microglia and in *in vivo* models of PD (Ahmed et al., 2021). Quercetin (Qu), a health supplement exerting anti-inflammatory and antioxidant effects, was shown to prevent mtROS accumulation and attenuate NLRP3 inflammasome activation and IL-1β release by promoting mitophagy in microglia, which ultimately alleviated the loss of DA neurons in PD mice (Han et al., 2021b). Qiu et al. (2022) found that UA mitigated NLRP3 inflammasome activation in LPS-stimulated BV2 cells and an MPTP-induced PD mouse model at least partly through the enhancement of microglial mitophagy, as disruption of microglial mitophagy with pharmacologic or genetic approaches impaired the neuroprotective effects of UA in PD.

### Ischemic stroke

Ischemic stroke is one of the most common health- and life-threatening diseases worldwide and is mainly caused by stenosis or obstruction of cerebral blood vessels. Mitophagy deficits are closely linked to the pathogenesis of ischemic stroke. Yuan et al. (2017) found that BNIP3L/NIX-mediated mitophagy, independent of PARK2 (Parkin-encoding gene), protected against cerebral ischemia. *BNIP3L knockout* impaired mitophagy and aggravated cerebral ischemia−reperfusion injury in mice, which was rescued by *BNIP3L overexpression*. Moreover, proteasome-mediated degradation of BNIP3L/NIX led to mitophagy deficiency in ischemic brains of mice (Wu et al., 2021). Another group clarified the role of microglial mitophagy during cerebral ischemic stroke (Han et al., 2021a). They found that microglial PGC-1α (a master coregulator of mitochondrial biogenesis) expression was rapidly increased in patients with ischemic stroke and middle cerebral artery occlusion (MCAO) mice, coinciding with an immediate response of microglial activation and inflammation after stroke. Microglia-specific overexpression of PGC-1α promoted mitophagy activity in microglia and ameliorated the inflammatory response and neurological deficits caused by ischemic injury in mice. Pharmacological or genetic knockdown of *Ulk1* blunted macroautophagy and mitophagy and abolished the neuroprotection elicited by microglial *PGC-1*α overexpression. Therefore, microglial mitophagy may be a promising therapeutic target for acute ischemic stroke. Nevertheless, microglia may produce diverse effects in the acute and chronic stages of ischemic stroke. The relevance of microglial mitophagy in tissue repair after stroke still needs to be explored.

### Other neurological disorders

Protein aggregate formation is a common characteristic of many neurodegenerative diseases. Mounting evidence suggests that impairment of the autophagy−lysosome pathway and selective mitophagy also serve as the common mechanism for these disorders, including amyotrophic lateral sclerosis (ALS) and Huntington’s disease (HD) (Martinez-Vicente et al., 2010; Sun et al., 2015; Moore and Holzbaur, 2016). However, current studies concentrate on the regulation of mitophagy in neuronal function and survival. For instance, the ALS-associated protein TDP-43 was reported to interact with the mitophagy receptor PHB2 and mitofusin 2 (MFN2) (Davis et al., 2018). ALS-linked mutations in *TBK1 or OPTN* can interfere with mitophagy and ultimately lead to neurodegeneration (Moore and Holzbaur, 2016). Similarly, mutations in *Huntingtin (HTT)* gene produced an inhibitory effect on mitophagy, resulting in an accumulation of damaged mitochondria and oxidative stress (Franco-Iborra et al., 2021). This was supported by the findings that morphologically abnormal mitochondria were found in neurons with mitophagy defects in HD flies and striatal cells of *HdhQ111* knock-in mice, which could be counteracted by *PINK1* overexpression (Khalil et al., 2015). To date, there is little evidence reporting the role of microglial mitophagy in ALS or HD.

In addition to neurodegeneration, microglial mitophagy is also linked to other neurological disorders. HIV-1-associated neurocognitive disorders (HANDs) constitute great challenges for human immunodeficiency virus-1 (HIV-1)-infected individuals, although the advent of combination antiretroviral therapy (cART) has dramatically increased their life expectancy. The cytotoxic HIV-1 protein, transactivator of transcription (TAT), is found to persist in the CNS and activate glial cells despite cART. Thangaraj et al. (2018) reported that HIV-1 TAT activated microglia and triggered neuroinflammatory responses by inducing the accumulation of damaged mitochondria due to mitophagy impairment. Similarly, this group demonstrated that a physiologically relevant dose of cocaine (10 μM) resulted in mitochondrial dysfunction and mitophagy deficiency in microglia, which contributed to microglial activation and proinflammatory cytokine generation (Thangaraj et al., 2020). Thus, manipulations that alleviate mitochondrial dysfunction or mitophagy defects may represent a therapeutic approach for these neurological disorders.

## Pharmacological regulation of mitophagy

At present, several natural compounds or drugs have been found to induce mitophagy in different tissues or cells. Table 1 summarizes the mechanisms and outcomes of these mitophagy-inducing compounds.

**TABLE 1:** Mechanisms and outcomes of mitophagy-inducing compounds.

Compounds	Mechanisms	Outcomes	References
	SIRT1-dependent deacetylation of Atg5, Atg7 and Atg8	Nutrient stress (−) Damaged mitochondria (−)	Lee et al. (2008)
	
NAD^+^ precursor (NR, NMN, NAM)
	DCT-1(NIX), PINK1, PDR1(Parkin) and p-ULK1 (+)	Cognitive decline (−) Prolong the Lifespan	Fang et al. (2019a,b)
	
	Nucleocytoplasmic transport of LC3 (+)	Autophagosome formation (+)	Huang et al. (2015)

	PINK-1, parkin, OPTN, p-Ulk1 (555), Beclin-1 and AMBRA1 (+)	Inflammation (−) Cognitive decline (−) Aβ/Tau pathology (−)	Fang et al. (2019b)
	
	Akt/mTOR signaling (−)	Inflammation (−)	Boakye et al. (2018)
Urolithin A
	
	Transcription factor EB activity (+)	Mitochondrial stress (−)	Tan et al. (2019)
	
	SIRT1-mediated deacetylation (+)	Inflammation (−) Aβ pathology (−)	Velagapudi et al. (2019)

	p-AMPK, Beclin-1 and LC3 (+)	Delay aging Inflammation (−) Cognitive decline (−)	Xu et al. (2020)
Spermidine
	
	PINK1/Parkin-dependent mitophagy	Cognitive decline (−) Health and lifespan (+) Locomotor capacity (+)	Yang et al. (2020)

	PINK1/Parkin-independent mitophagy	Damaged mitochondria (−)	Allen et al. (2013)
Deferiprone
	
	Mitochondrial ferritin (+)	Hepatocellular carcinoma (−)	Hara et al. (2020)

Metformin	PINK1/Parkin-mediated mitophagy	Osteoarthritis-like inflammation (−) Liver injury (−)	Song et al. (2016), Wang et al. (2019)

(−), inhibition or decrease; (+), promotion or increase.

### NAD^+^

NAD^+^, also known as coenzyme I, is a metabolic intermediate in the respiratory electron transport chain and plays an essential role in maintaining mitochondrial homeostasis and genome stability. A growing number of studies have reported a decrease in intracellular NAD^+^ levels during aging and age-related diseases (Verdin, 2015). NAD^+^ can promote healthy aging by regulating mitochondrial biogenesis and mitophagy (Fang et al., 2014; Vannini et al., 2019). NAD^+^ precursors [(NR, nicotinamide nucleotide (NMN, nicotinamide mononucleotide) and (NAM, nicotinamide)] were shown to prolong the lifespan in worms, Drosophila, and rodent models (Canto et al., 2015; Yaku et al., 2018; Yoshino et al., 2018; Lautrup et al., 2019), indicating a conserved antiaging function of NAD^+^ across different species.

NAD^+^ levels were decreased in early onset familial AD patients and Aβ-stimulated rat cortical neurons. Enhancement of NAD^+^ was neuroprotective against AD pathology and cognitive deficits. In AD amyloidosis models, supplementation with the NAD^+^ precursors NMN and NR improved cognitive impairment and reduced Aβ plaque formation in both nematode and mouse AD models (Gong et al., 2013; Sorrentino et al., 2017; Fang et al., 2019b). NAD^+^ also inhibited inflammasome activation and tau aggregation and rescued cognitive decline in 3 × Tg-AD mice (Hou et al., 2018). All these effects are dependent on the regulation of mitophagy and mitochondrial function by NAD^+^.

Microglial activation and neuroinflammation participate in neurodegeneration. However, it remains unclear whether NAD^+^ exerts antiaging or neuroprotective effects *via* a direct effect on neurons or an indirect impact on microglia. Most studies have reported that NAD^+^ promotes mitophagy in neurons and prevents neuronal death (Fang et al., 2016; Aman et al., 2020). Mitophagy disruption occurs not only in neurons but also in microglia (Hou et al., 2021). Treatment with the NAD^+^ precursor NMN/NR was shown to inhibit microglia-induced neuroinflammation and improve cognitive function in disease models other than AD (Zhao et al., 2021). However, it remains to be answered whether neuronal and microglial mitophagy contribute equally to the neuroprotective actions of NAD^+^.

How does NAD^+^ induce mitophagy? As a cofactor, NAD^+^ serves as the substrate of various enzymes, including sirtuins/SIRTs, PARPs/poly (ADP-ribose polymerases) and cyclic ADP-ribose synthases (CD38, BST1/CD157). Therefore, NAD^+^ supplementation can trigger autophagy or mitophagy by affecting different signaling pathways. For example, SIRT1 utilizes NAD^+^ to deacetylate autophagy-related proteins, and loss of *SIRT1* inhibits autophagy (Lee et al., 2008; Huang et al., 2015). NAD^+^/SIRT1 pathway activation restored mitophagy by upregulating DCT-1 (the nematode BNIP3 and BNIP3L/NIX homolog) and activating Ulk1 (Fang et al., 2019a,b). The membrane-bound NADase CD38, which hydrolyzes NAD^+^ to nicotinamide and cyclic ADP-ribose, plays a crucial role in autophagosome fusion with lysosomes (Xiong et al., 2013). Additionally, the PARP family can bind to the circulating ADP-ribose synthase CD38 and result in NAD^+^ depletion, thereby limiting SIRT1 activity and inhibiting mitophagy (Aman et al., 2020). Overall, NAD^+^ can regulate mitophagy through multiple pathways. The presence of natural NAD^+^ precursors and their safety and efficacy in clinical trials make them potential candidates for mitophagy targeting. However, it is unmet to gain an in-depth understanding of NAD^+^ precursor treatment on cognitive functions for antiaging and neuroprotection.

### Urolithin A

UA is a natural metabolite produced by gut flora from ellagitannins in pomegranate fruits, nuts and berries. It induced mitophagy in *C. elegans*, mammalian cells and rodents (Ryu et al., 2016). UA and AC were shown to relieve Aβ and tau pathologies by enhancing neuronal mitophagy and reversing memory loss in transgenic tau nematodes and 3 × TgAD mice (Fang et al., 2019b). Increased engulfment of Aβ plaques by microglia was observed in UA- and AC-treated APP/PS1 mice (Fang et al., 2019b). This finding coincided with the decrease in proinflammatory cytokines in the isolated microglia from the hippocampus of UA-treated AD mice. Consistently, another study reported that UA protected against DA neuron degeneration and NLRP3 inflammasome-related neuroinflammation *via* the enhancement of microglial mitophagy in an MPTP-induced PD mouse model (Qiu et al., 2022). More importantly, a first-in-human clinical trial showed that UA treatment, either as a single dose or as multiple doses at 500 and 1,000 mg for 4 weeks, modulated plasma acylcarnitine and skeletal muscle mitochondrial gene expression, implicating a favorable safety profile of the UA regimen for the improvement of muscle strength and physical performance in elderly individuals. This represents a translational potential of UA in helping control mitochondrial dysfunction-related diseases and aging (Andreux et al., 2019).

Fang et al. (2019b) reported that UA induced mitophagy by upregulating a series of mitophagy-related proteins, including PINK-1, parkin, OPTN, p-Ulk1 (555), Beclin-1, Bcl2L13, and AMBRA1. In addition, Boakye et al. (2018) reported that UA enhanced the autophagic flux by inhibiting Akt/mTOR signaling and played an anti-inflammatory role in LPS-stimulated macrophages. The pomegranate extract, a UA precursor, was shown to facilitate mitophagy by modulating transcription factor EB (TFEB) activity in stressed SH-SY5Y cells (Tan et al., 2019). A SIRT-1-dependent autophagy induction was also involved in UA-elicited neuroprotection in brain cells (Velagapudi et al., 2019). However, the exact signaling cascade for UA-induced mitophagy in microglia is poorly understood, and whether mitophagy enhancement is responsible for its anti-neuroinflammatory functions also warrants further study.

### Spermidine

Spermidine is a natural polyamine present in all living organisms and a variety of foods, including mature cheeses, beans, and cereals. Spermidine has antiaging, anti-inflammatory, and neuroprotective properties. Remarkably, intracellular spermidine levels decline with age (Eisenberg et al., 2009). Supplementation with spermidine markedly extended the lifespan of yeast, flies and worms *via* the alteration of chromatin acetylation status and upregulation of autophagy-related genes. A recent study demonstrated that spermidine had an antiaging effect in *d*-galactose (d-Gal)-treated mouse neuroblastoma (N2a) cells by improving autophagy and mitochondrial genome stability (Jing et al., 2018). In addition, spermidine inhibited memory impairment in AD worms and improved motor performance in a PD worm model *via* PINK1-PDR1 (the nematode PRKN)-dependent mitophagy (Yang et al., 2020), and also showed benefits on cognitive function in animal models. However, the negative results of a clinical trial for spermidine against cognitive decline in older population were recently published (Schwarz et al., 2022). Regulation of AMPK/mTOR signaling was shown to be responsible for spermidine and spermine -induced mitophagy (Xu et al., 2020). To date, few studies have explored the impact of spermidine on microglial mitophagy. In fact, an anti-inflammatory effect of spermidine was reported in LPS-stimulated BV2 microglial cells (Choi and Park, 2012). However, whether this was attributable to mitophagy regulation remains unknown.

### Others

In recent years, other compounds have been reported to modulate mitophagy. For instance, the iron chelator deferiprone induced mitophagy through a PINK1/PARKIN-independent pathway (Allen et al., 2013; Hara et al., 2020). Metformin, an antidiabetic drug, also induced mitophagy independent of glucose-lowering effect (Zheng et al., 2012; Song et al., 2016; Wang et al., 2019). Rapamycin and resveratrol prolonged the lifespan of various organisms by triggering mitophagy (Ren and Zhang, 2018; Li et al., 2020). Curcumin, a well-known polyphenolic compound, was shown to protect against cerebral ischemia−reperfusion injury by enhancing mitophagy and preserving mitochondrial dynamics (de Oliveira et al., 2016; Wang and Xu, 2020).

Remarkably, some proton carriers, such as CCCP, FCCP, and DNP, which are widely used in cell biology research, were shown to induce mitophagy *in vitro*. However, traditional proton carriers are not specific for mitochondria and have proton-promoting activity on other biological membranes (Georgakopoulos et al., 2017), which often results in high toxicity and a narrow therapeutic dose range. Thus, the translational potential in clinical use is limited. Moreover, excess mitochondria uncoupling in neurons may cause ATP decline and even neuronal cell death (Childress et al., 2018). Hence, novel microglia-specific mitochondrial uncouplers can be developed to induce mitophagy and alleviate mitochondrial dysfunction- or neuroinflammation-related disorders.

Interestingly, dietary or caloric restriction appears to be an effective approach to improve mitochondrial turnover *via* mitophagy in aging organisms (Choi et al., 2013; Weir et al., 2017; Mooli et al., 2020). Physical exercise also represents a natural means of triggering mitophagy (Drake et al., 2021). Yet, it remains unknown whether microglial mitophagy is affected by these lifestyle changes.

## Summary and perspectives

The brain is an organ with a high energy demand, and homeostasis maintenance of the mitochondrial network is crucial for energy supply and brain function. Aberrant accumulation of damaged mitochondria, along with mitophagy defects, is a critical contributor to various neurological disorders. Microglia, the brain resident innate immune cells, participate in brain development and homeostasis maintenance throughout life. Microglia-mediated neuroinflammation is intimately linked to neurodegeneration and other neurological disorders. A number of studies have demonstrated a beneficial role of mitophagy in suppressing microglia-mediated neuroinflammation by reducing mtROS generation and inhibiting the NLRP3 inflammasome and cGAS/STING pathway. A few studies have also revealed an impact of mitophagy on the phagocytic ability of microglia, although the underlying mechanism is unclear. Mitophagy-mediated removal of dysfunctional mitochondria and improvement of mitochondrial biogenesis and dynamics may be involved. All in all, microglial mitophagy may become a promising target for the treatment of aging and inflammation-related neurological disorders. To date, limited approaches, based on pharmacologic and non-pharmacologic interventions, have been reported to enhance microglial mitophagy and thus exert neuroprotection *in vitro* and *in vivo*. How to specifically enhance microglial mitophagy but not induce overwhelming mitophagy or autophagic death in neurons remains an open question. More importantly, current studies reveal a role for mitophagy and its regulatory factors in removing depolarized or damaged mitochondria under stressful conditions. Relatively less is known regarding the machinery of basal mitophagy *in vivo*. Distinct basal and stress-evoked mitophagy pathways may orchestrate mitochondrial clearance in a context-dependent fashion. Whether basal mitophagy affects the physiological function of microglia during brain development and the adult stage also needs to be investigated. There is still a long way to go for a comprehensive understanding in the role and regulation of microglial mitophagy in the brains of both healthy and diseased individuals.

## Author contributions

YL, MW, and X-OH conceived and drafted the original manuscript and designed the figures. X-OH revised and edited the manuscript. L-FH supervised the draft and made critical revisions to the manuscript. All authors contributed to the article and approved the submitted version.

## Abbreviations

AC: actinonin
AD: Alzheimer’s disease
ALS: amyotrophic lateral sclerosis
AMBRA1: autophagy and Beclin 1 regulator 1
ATP: adenosine triphosphate
A β: amyloid β
BNIP3: BCL2/adenovirus E1B 19-kDa-interacting protein 3
BNIP3 L/NIX: BCL2/adenovirus E1B 19-kDa-interacting protein 3-like
CNS: central nervous system
DUBs: deubiquitinating enzymes
FUNDC1: FUN14 domain-containing 1
HD: Huntington’s disease
HIV-1: human immunodeficiency virus-1
HTT: Huntingtin
IMM: inner mitochondrial membrane
MAVS: mitochondrial antiviral signaling
mtDNA: mitochondrial DNA
mtROS: mitochondrial reactive oxygen species
NMN: nicotinamide mononucleotide
NR: nicotinamide nucleotide
OMM: outer mitochondrial membrane
OPTN: optic nerve protein
PD: Parkinson’s disease
PHB2: prohibitin 2
PINK1: PTEN-induced putative kinase protein 1
PLS3: phospholipid scramblase-3
TBK1: TANK binding kinase 1
Ub: ubiquitin
UA: urolithin A.
